# Human Induced Pluripotent Stem Cell Derived Neuronal Cells Cultured on Chemically-Defined Hydrogels for Sensitive *In Vitro* Detection of Botulinum Neurotoxin

**DOI:** 10.1038/srep14566

**Published:** 2015-09-28

**Authors:** Sabine Pellett, Michael P. Schwartz, William H. Tepp, Richard Josephson, Jacob M. Scherf, Christina L. Pier, James A. Thomson, William L. Murphy, Eric A. Johnson

**Affiliations:** 1 Department of Bacteriology, University of Wisconsin at Madison, Madison, Wisconsin, United States of America; 2 Department of Biomedical Engineering, University of Wisconsin at Madison, Madison, Wisconsin, United States of America; 3 MTI-GlobalStem, Inc. Gaithersburg, Maryland, United States of America; 4 Department of Cell and Regenerative Biology, University of Wisconsin at Madison, Madison, Wisconsin, United States of America; 5 Morgridge Institute for Research, Madison, Wisconsin, United States of America; 6 Department of Molecular, Cellular, and Developmental Biology, University of California at Santa Barbara, Santa Barbara, California, United States of America; 7 Department of Orthopedics and Rehabilitation, University of Wisconsin at Madison, Madison, Wisconsin, United States of America

## Abstract

Botulinum neurotoxin (BoNT) detection provides a useful model for validating cell-based neurotoxicity screening approaches, as sensitivity is dependent on functionally competent neurons and clear quantitative endpoints are available for correlating results to approved animal testing protocols. Here, human induced pluripotent stem cell (iPSC)-derived neuronal cells were cultured on chemically-defined poly(ethylene glycol) (PEG) hydrogels formed by “thiol-ene” photopolymerization and tested as a cell-based neurotoxicity assay by determining sensitivity to active BoNT/A1. BoNT/A1 sensitivity was comparable to the approved *in vivo* mouse bioassay for human iPSC-derived neurons and neural stem cells (iPSC-NSCs) cultured on PEG hydrogels or treated tissue culture polystyrene (TCP) surfaces. However, maximum sensitivity for BoNT detection was achieved two weeks earlier for iPSC-NSCs that were differentiated and matured on PEG hydrogels compared to TCP. Therefore, chemically-defined synthetic hydrogels offer benefits over standard platforms when optimizing culture conditions for cell-based screening and achieve sensitivities comparable to an approved animal testing protocol.

There is growing concern over a possible link between neurodevelopmental disorders and exposure to chemicals in the environment^1^,^2^,^3^, and even subtle neurotoxic effects on cognitive function may have substantial consequences to society when extrapolated to the population level^4^. Despite these potential risks, few chemicals have been extensively evaluated for neurotoxicity^1^,^2^,^3^, largely due to limited predictive value^5^, prohibitive cost, and ethical considerations associated with animal testing^1^,^2^,^3^. In 2007, the National Research Council (NRC) published a vision for the future of toxicity screening and pathway analysis that is focused on strategies to reduce animal testing by implementing human cell-based models^6^. While cell-based assays are a priority for regulatory agencies, *in vitro* approaches must meet strict quality control guidelines and will require validation before replacing animal testing for toxicity screening and safety assessment^1^,^2^,^7^. A major challenge towards validation of cell-based assays for assessing neurotoxicity is a limited understanding of mechanisms of action specific to human neurophysiology, and benchmarks for justifying the replacement of animal testing are not clearly established for most *in vitro* approaches^1^,^2^,^3^.

Botulinum neurotoxin (BoNT) detection provides a well-defined model for testing cell-based neurotoxicity assays^8^, as sensitivity is dependent on functionally competent neurons and clear quantitative endpoints are available for comparing against the approved safety assessment model, the *in vivo* mouse bioassay^8^,^9^,^10^,^11^,^12^,^13^,^14^,^15^,^16^,^17^. The BoNTs are the most potent known human toxins, exerting their toxicity by entering neuronal cells of the peripheral nervous system and blocking neurotransmitter release at the neuromuscular junction^18^, with a parenteral human lethal dose estimated to be as low as 1 ng/kg^19^. BoNTs are 150 kDa protein toxins consisting of a 100 kDa heavy chain and 50 kDa light chain linked by a disulfide bond. Cell entry proceeds via a series of consecutive and essential steps that result in cleavage of the disulfide bond to release the light chain into the cytosol, where it is refolded into the enzymatically active form^20^,^21^,^22^,^23^. The active BoNT light chain cleaves the soluble N-ethylmaleimide-sensitive-factor attachment receptor (SNARE) protein family, which is an essential component of neurotransmitter release^24^,^25^,^26^. Researchers have taken advantage of this mechanistic framework to develop *in vitro* cell-based assays that identify active BoNTs with sensitivities that are comparable to the mouse bioassay^8^,^9^,^10^,^11^,^12^,^13^,^14^,^15^,^16^, including the first to be approved by the FDA for safety assessment of a pharmaceutical BoNT/A1 product^17^.

The aim of the present study was to establish a robust neurotoxicity screening assay suitable for standardization by using a scalable, non-cancerous human cell source and a chemically-defined culture substrate. Synthetic poly(ethylene glycol) (PEG) hydrogels formed by “thiol-ene” photopolymerization^27^ were chosen as a chemically defined culture substrate due to the versatility of this platform for modeling diverse cell functions^28^,^29^,^30^,^31^,^32^,^33^,^34^,^35^,^36^,^37^,^38^,^39^,^40^,^41^. Human pluripotent stem cells provide a uniform and expandable source for tissue-specific cell types^42^,^43^,^44^, including diverse neural and glial phenotypes^45^,^46^,^47^,^48^. Therefore, human induced pluripotent stem cell (iPSC)-derived neuronal cells were chosen as the cellular component for the neurotoxicity screening assay here. A particular emphasis of the present study was to explore the potential for iPSC-derived neural stem cells (iPSC-NSCs) as the cellular component for neurotoxicity screening, since these cells are expandable and can be differentiated down multiple neuronal and glial lineages, and thus offer greater flexibility towards optimizing neural phenotypes for specific cell-based applications^49^,^50^. Finally, active botulinum neurotoxin A1 (BoNT/A1) was chosen as a model toxin for validating human iPSC-derived neuronal cells cultured on PEG as a neurotoxicity assay, since this serotype has been adapted for a variety of pharmacological applications^51^ and can be detected with high sensitivity using functional neuronal cells^8^,^9^,^10^,^11^,^12^,^13^,^14^,^15^,^16^,^17^.

## Results and Discussion

**Figure 1:**
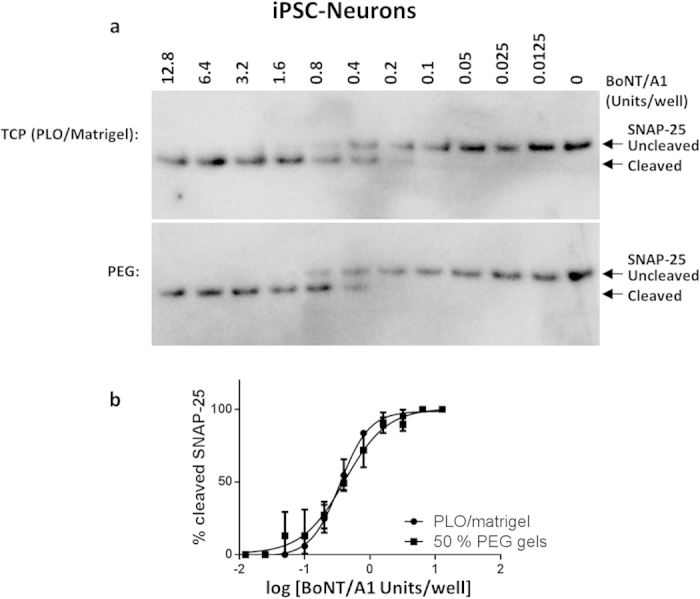
BoNT/A1 detection using human iPSC-neurons cultured on TCP or PEG hydrogel surfaces. Representative **(a)** Western blots for SNAP-25 cleavage and **(b)** EC50 curves for human iPSC-neurons cultured on poly-L-ornithine and Matrigel (PLO/Matrigel) coated tissue culture polystyrene (TCP) or poly(ethylene glycol) (PEG) hydrogels and treated with serial dilutions of BoNT/A1 (Units/Well). iPSC-neurons were exposed to BoNT/A1 for 48 h before cell lysates were harvested, followed by Western blot and densitometry analysis to quantify SNAP-25 cleavage. Sensitivity is expressed as BoNT activity (Units/well) to reach half the maximum response (EC50) for SNAP-25 cleavage, where 1 U is equivalent to the mLD50 determined using an *in vivo* mouse bioassay. The EC50 for iPSC-neurons was 0.41 ± 0.04 U/well on PEG hydrogels and 0.38 ± 0.06 U/well on PLO/Matrigel coated TCP.

BoNT/A1 was previously detected with sensitivity that exceeded the *in vivo* mouse bioassay using iPSC-derived neurons (iPSC-neurons) cultured on poly-L-ornithine and Matrigel (PLO/Matrigel) coated tissue culture polystyrene (TCP)^11^. Therefore, BoNT/A1 detection was first compared for iPSC-neurons cultured on PEG hydrogels and PLO/Matrigel coated TCP surfaces to determine a baseline for sensitivity relative to the established assay^11^. PEG hydrogels were formed by crosslinking 8-arm PEG-norbornene molecules with PEG-dithiol molecules^29^, while pendant CRGDS peptide was incorporated to promote cell adhesion^52^ (see Methods). For *in vitro* cell-based assays, sensitivity is usually expressed as Units (U) of BoNT activity to reach half the maximum response (EC50) for SNAP-25 cleavage (Quantified by western blot as described in Methods; Fig. 1). The potency of BoNT/A1 preparations is established using the *in vivo* mouse bioassay, where 1 U is equivalent to the dose that leads to 50% lethality in mice within a four day period after intraperitoneal injection (mouse lethal dose 50, mLD50)^53^,^54^. The sensitivities for BoNT/A1 detection by iPSC-neurons (Fig. 1) cultured on both PEG hydrogels (0.41 ± 0.04 U/well) and PLO/Matrigel coated TCP (0.38 ± 0.06 U/well) exceeded the *in vivo* mouse bioassay and were comparable to previous benchmarks established in the literature (~0.1–1 U)^10^,^11^.

**Figure 2:**
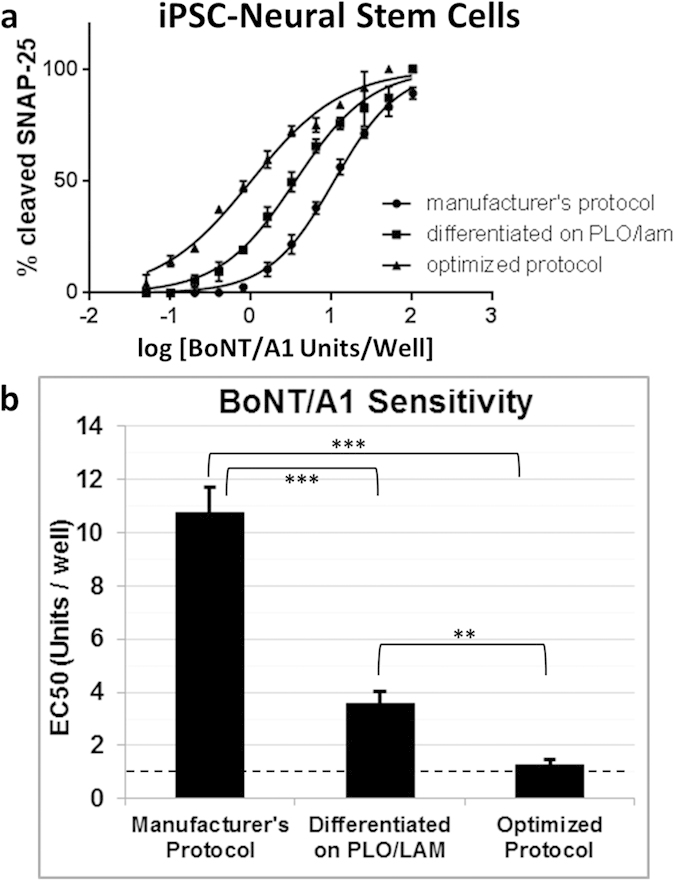
Optimization of BoNT/A1 sensitivity for human iPSC-NSCs differentiated and matured on TCP. The manufacturer’s protocol (see Methods) was modified to optimize BoNT/A1 sensitivity for iPSC-NSCs cultured on TCP by replacing the proprietary pre-coat solution with poly-L-ornithine and laminin treatment (“Differentiated on PLO/LAM”), supplementing the differentiation medium with retinoic acid (RA) and purmorphamine (PUR), and extending the maturation time to 23 days (“Optimized Protocol”). **(a)** Representative EC50 curves for human iPSC-neural stem cells (iPSC-NSCs) treated with serial dilutions of BoNT/A1 (Units/Well). Cultured cells were exposed to BoNT/A1 for 48 h before cell lysates were harvested, followed by Western blot and densitometry analysis to quantify SNAP-25 cleavage. **(b)** BoNT/A1 sensitivities (EC50, Mean ± S.D., 3 replicate experiments) for iPSC-NSCs cultured on TCP surfaces. The EC50 value is defined as the BoNT activity (Units/Well) required to reach half the maximum response for SNAP-25 cleavage, where 1 U is equivalent to the mLD50 determined using an *in vivo* mouse bioassay (dashed line). Statistical significance was determined using a one-way ANOVA (alpha = 0.05) followed by a Tukey test to compare individual means (Multiplicity adjusted P-values: **P ≤ 0.01; ***P ≤ 0.001).

**Figure 3:**
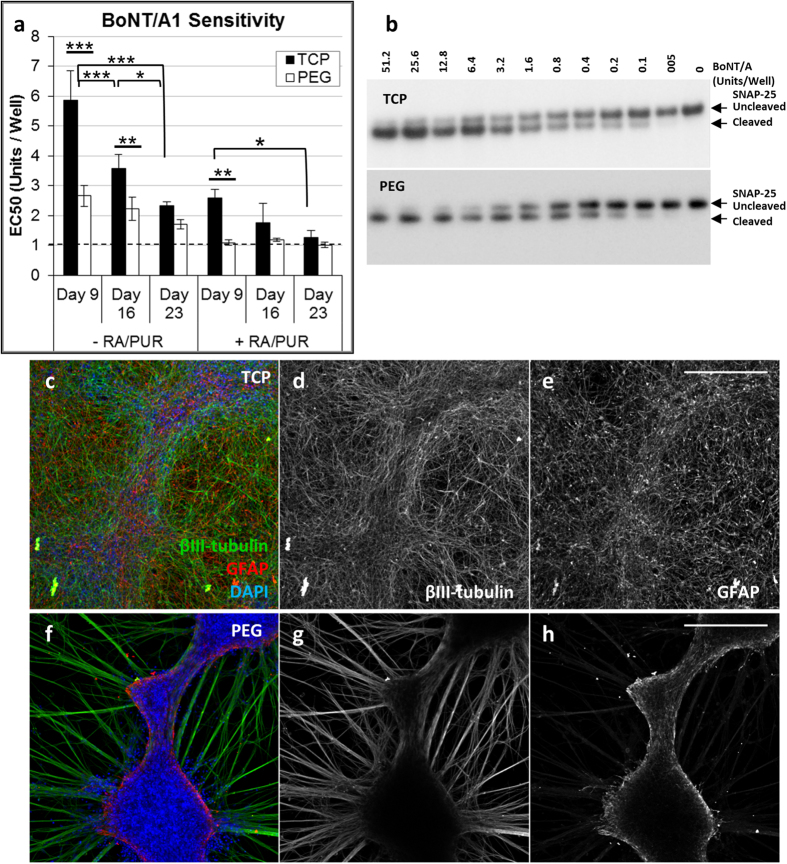
Morphologies and BoNT/A1 sensitivity for iPSC-NSCs cultured on TCP or PEG hydrogel surfaces. A comparison of iPSC-NSCs cultured on PEG hydrogels (PEG) or PLO/LAM coated TCP surfaces (TCP), differentiated for 5 days with (+) or without (−) RA/PUR and matured for 9, 16, or 23 days. **(a)** BoNT/A1 sensitivities (EC50, Mean ± S.D., 3 replicate experiments) for iPSC-NSCs cultured on TCP or PEG surfaces. The EC50 value is defined as BoNT activity (Units/Well) required to reach half the maximum response for SNAP-25 cleavage, where 1 U is equivalent to the mLD50 determined using an *in vivo* mouse bioassay (dashed line). Statistical significance was determined using a one-way ANOVA (alpha = 0.05) followed by a Tukey test to compare individual means (Multiplicity adjusted P-values: *P ≤ 0.05; **P ≤ 0.01; ***P ≤ 0.001) See Supplementary Table S1 for all sample comparisons. **(b)** Western blot data showing SNAP-25 cleavage for iPSC-NSCs that were differentiated for 5 days (with RA/PUR) and matured for 23 days and then treated with serial dilutions of BoNT/A1 (Units/Well). **(c–h)** Immunofluorescence imaging illustrating **(c,f)** βIII-tubulin (neurons, green), GFAP (glial, red), and DAPI (nuclei, blue) expression and single channel grayscale images for **(d,g)** βIII-tubulin, and **(e,h)** GFAP. Human iPSC-NSCs were differentiated (+RA/PUR) and matured (23 days) on **(c–e)** PLO/LAM treated TCP and **(f–h)** PEG hydrogels with 50% non-degradable SH-PEG-SH crosslinks and 3 mM CRGDS. **Scale Bars**: 250 μm.

While iPSC-neurons have been established for detecting BoNTs with sensitivity that exceeds the mouse bioassay^11^, these cells are terminally differentiated and post-mitotic, which introduces high costs and may be limiting for applications that require other neuronal phenotypes. Therefore, BoNT/A1 detection was optimized using human iPSC-derived neural stem cells (iPSC-NSCs) to provide an alternative cell source that is expandable and which can be differentiated down multiple neuronal lineages. The EC50 for BoNT/A1 detection was 10.7 ± 0.9 U/well when iPSC-NSCs were differentiated and matured on TCP using the manufacturer’s protocol (Fig. 2; “Manufacturer’s Protocol”; See Methods for details), which is substantially lower than the sensitivity achieved for iPSC-neurons (Fig. 1)^11^. Therefore, we aimed to enhance BoNT/A1 sensitivity for iPSC-NSCs cultured on TCP by exploring the effects of surface coatings, differentiation factors, and maturation times. Replacing the manufacturer’s proprietary pre-coat solution with poly-L-ornithine (PLO) and laminin (Fig. 2; “Differentiated on PLO/LAM”) treatment improved sensitivity for BoNT/A1 detection from 10.7 ± 0.9 U/well to 3.6 ± 0.5 U/well, with similar results on PLO/Matrigel coated surfaces (such as used for iPSC-neurons). Both PLO/LAM and PLO/Matrigel surface coatings have been applied to BoNT assays, but Matrigel is composed of a mixture of poorly-defined bioactive components, including many potent growth factors known to influence stem cell fate^55^. Therefore, PLO/LAM treatment was used for subsequent experiments on TCP to provide a better defined surface. Differentiation in Neurobasal medium supplemented with B27, glutamax, cyclic adenosine monophosphate (cAMP), brain-derived neurotrophic factor (BDNF), or glial cell-derived neurotrophic factor (GDNF) did not improve BoNT/A1 sensitivity. However, sensitivity improved from 3.6 ± 0.5 U/well to 1.8 ± 0.7 U/well for iPSC-NSCs that were differentiated in the presence of retinoic acid (RA) and purmorphamine (PUR)^56^. Finally, the highest sensitivity for BoNT/A1 detection (1.3 ± 0.2 U/well) was achieved for iPSC-NSCs that were matured for 23 days (Fig. 3a). Thus, BoNT/A1 detection was increased for iPSC-NSCs cultured on TCP surfaces coated with PLO/LAM, differentiated in medium supplemented with RA and PUR, and matured for 23 days (Fig. 2; “Optimized Protocol”).

BoNT/A1 detection was then compared for iPSC-NSCs differentiated and matured on PEG hydrogels and PLO/LAM coated TCP surfaces. BoNT/A1 sensitivity for iPSC-NSCs differentiated and matured on PEG hydrogels minimally depended on matrix properties within the limited range of conditions explored (Supplementary Fig. S1), which included different crosslinking densities and RGD adhesion ligand concentrations^52^, as well as substitution of the non-degradable PEG-dithiol crosslinker with a protease-degradable peptide (Supplementary Fig. S1, Supplementary Table S1)^57^. Therefore, subsequent comparisons to TCP were performed using PEG hydrogels with a non-degradable PEG-dithiol crosslinker, 50% crosslinking density, and 3 mM CRGDS. BoNT/A1 sensitivity was improved when iPSC-NSCs were differentiated in medium supplemented with RA and PUR for both TCP and PEG hydrogels (Fig. 3a, “-RA/PUR” vs. “+RA/PUR”). A trend for increasing BoNT/A1 sensitivity with longer maturation time was repeatedly observed for iPSC-NSCs cultured on TCP, with the highest sensitivity of 1.3 ± 0.2 U/well achieved after 23 days (Fig. 3a, “TCP”, +RA/PUR condition). When iPSC-NSCs were differentiated and matured for 23 days on PEG hydrogels, sensitivity for BoNT/A1 detection (EC50 = 1.0 ± 0.1 U/well, Fig. 3a) and SNAP-25 cleavage patterns (Fig. 3b) were comparable to TCP. However, a similar EC50 of 1.1 ± 0.1 U/well was achieved for iPSC-NSCs cultured on PEG hydrogels after only nine days of maturation (Fig. 3a, “PEG”, +RA/PUR condition). Thus, iPSC-NSCs required approximately 1–2 weeks less maturation time to detect BoNT/A1 with sensitivity equivalent to the *in vivo* mouse bioassay when cultured on PEG hydrogels compared to PLO/LAM coated TCP.

**Figure 4:**
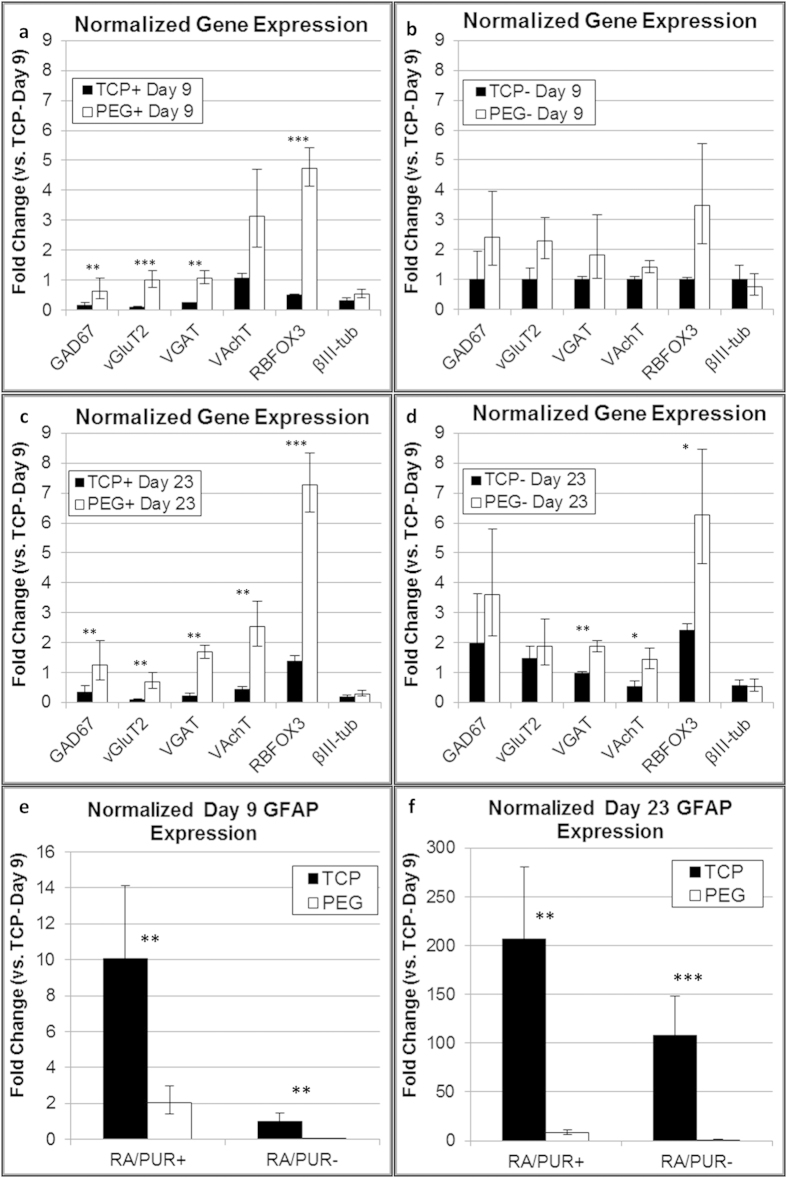
Gene expression for iPSC-ECs differentiated and matured on TCP or PEG hydrogel surfaces. Normalized gene expression was determined by RT-PCR for iPSC-NSCs cultured on PLO/LAM coated TCP (TCP) or poly(ethylene glycol) (PEG) hydrogel surfaces, differentiated with (+) or without (−) RA/PUR in the culture medium, and matured for **(a,b,e)** 9 days or **(c,d,f)** 23 days. Gene quantities were normalized to GAPDH and graphs represent fold-changes relative to iPSC-NSCs that were differentiated on TCP without RA/PUR and matured for 9 days (Mean ± S.D., n = 6 samples from 2 replicate experiments). Statistical significance was determined using a Student’s *t*-test (*P < 0.05; **P < 0.01; ***P < 0.005).

**Figure 5:**
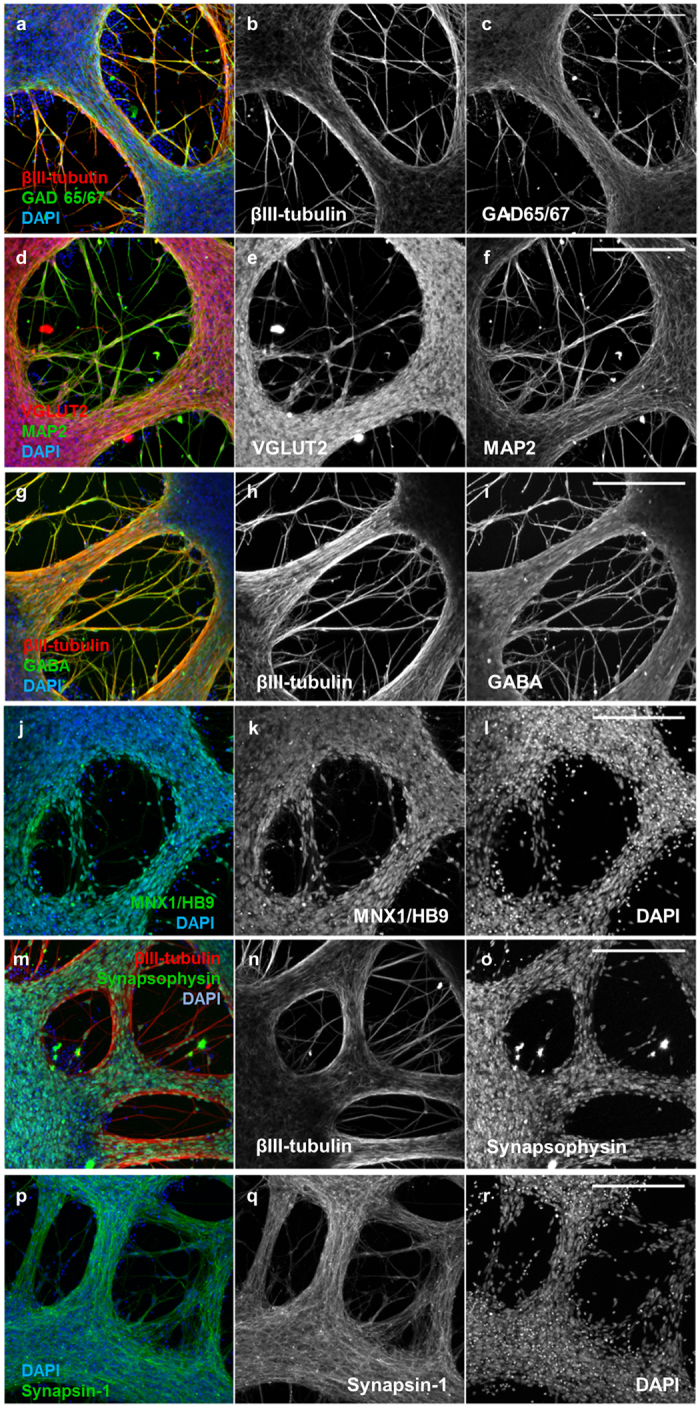
Neuronal markers expressed by iPSC-NSCs after differentiation and maturation on PEG hydrogels. **(a)** βIII-tubulin (red), GAD 65/67 (green), and DAPI (nuclei, blue). Single channel grayscale images from (a) are shown for **(b)** βIII-tubulin and **(c)** GAD 65/67. **(d)** VGLUT2 (red), MAP2 (green), and DAPI (nuclei, blue). Single channel grayscale images from (d) are shown for **(e)** VGLUT2 and **(f)** MAP2. **(g)** βIII-tubulin (red), GABA (green), and DAPI (nuclei, blue). Single channel grayscale images from (**g**) are shown for **(h)** βIII-tubulin and **(i)** GABA. **(j)** MNX1/HB9 (green) and DAPI (nuclei, blue). Single channel grayscale images from (**j**) are shown for **(k)** MNX1/HB9 and **(l)** DAPI. **(m)** βIII-tubulin (red), Synapsophysin (green), and DAPI (nuclei, blue). Single channel grayscale images from (m) are shown for **(n)** βIII-tubulin and **(o)** Synapsophysin. **(p)** Synapsin-1 (green) and DAPI (nuclei, blue). Single channel grayscale images from (p) are shown for **(q)** Synapsin-1 and **(r)** DAPI. **Scale Bars**: 200 μm.

We hypothesized that differences in differentiated cell populations generated by iPSC-NSCs on PEG hydrogels may play a role in the shorter maturation time required to reach maximum BoNT/A1 sensitivity compared to TCP. To test this hypothesis, immunofluorescence microscopy (Fig. 3c–h) and RT-PCR (Fig. 4) were performed to analyze neuronal and glial markers for iPSC-NSCs after differentiation and maturation on PEG hydrogels and PLO/LAM coated TCP surfaces. Immunofluorescence imaging identified mixed βIII-tubulin^+^ and GFAP^+^ populations on PEG hydrogels (Fig. 3f–h) and TCP surfaces (Fig. 3c–e) when iPSC-NSCs were differentiated and matured for 23 days (with RA/PUR), but morphological features differed substantially between the two culture platforms. Specifically, whereas iPSC-NSCs were characterized mostly by disorganized mesh-like layers after differentiation and maturation on TCP (Fig. 3c), cells cultured on PEG hydrogels formed organized clusters with dense bundles of interconnected neuronal processes (Fig. 3f). Further analysis by RT-PCR demonstrated that iPSC-NSCs were characterized by significantly higher gene expression for several neuronal markers when differentiated on PEG hydrogels (Fig. 4a–d), including RBFOX3, which encodes the NeuN epitope associated with maturity in many human neurons^58^. Consistent with RT-PCR results, iPSC-NSCs differentiated into neuronal cells with distinct phenotypes (e.g., glutamatergic and GABAergic neurons, Fig. 5a–l) and expressed markers for synaptic proteins (e.g., synapsin and synaptophysin, Fig. 5m–r) when cultured on PEG hydrogels. In contrast, GFAP was more highly expressed for iPSC-NSCs cultured on TCP regardless of condition, with >20-fold higher expression than cells cultured on PEG hydrogels by day 23, including cells differentiated with or without RA/PUR (Fig. 4e–f). Thus, iPSC-NSCs that were differentiated and matured on PEG hydrogels were characterized by enhanced expression for several neuronal markers and relatively lower expression of GFAP compared to cells cultured on TCP surfaces.

Previous studies have reported that glial phenotypes are enriched for primary rat neural progenitor cells cultured on stiffer substrates^59^,^60^, while enhanced neuronal differentiation has been observed for both rat^61^ and human pluripotent stem cells cultured on compliant surfaces^34^. A role for mechanical properties in directing neural stem cell function is notable, since the modulus for TCP (~GPa)^62^,^63^ is approximately six orders of magnitude higher than PEG hydrogels formed within the range of conditions reported here (~kPa)^29^, and compared to neural tissue *in vivo* (~kPa)^62^. GFAP gene expression was substantially upregulated for iPSC-NSCs on TCP relative to cells on PEG hydrogels, which would be consistent with enrichment of glial phenotypes on stiffer substrates. However, while GFAP expression is specific to mature glial phenotypes in rodents, it is expressed by human radial glia during neurogenesis^64^. Therefore, higher GFAP expression by iPSC-NSCs cultured on TCP may be due to maintenance of a progenitor state rather than enhanced differentiation into mature glial cell types (e.g., astrocytes or oligodendrocytes). Consequently, functional neurons may emerge earlier for iPSC-NSCs cultured on PEG hydrogels, a possibility that is supported by relatively higher gene expression for neuronal markers relative to cells cultured on TCP by nine days of maturation (Fig. 4). Despite differences in neural differentiation for iPSC-NSCs cultured on PEG hydrogels and TCP surfaces, there were no clear correlations to BoNT/A1 sensitivity for any of the markers investigated. Therefore, further investigation will be required to determine the molecular mechanisms that lead to higher BoNT/A1 sensitivity by day 9 of maturation when iPSC-NSCs are cultured on PEG hydrogels, including analysis of cell properties and potential effects of differences in cell culture (e.g., cell density or morphological characteristics). Nevertheless, our results highlight differences in differentiation for iPSC-NSCs cultured on PEG hydrogels and TCP surfaces that have broader implications for biomedical engineering and toxicology research.

Our combined results demonstrate that iPSC-NSCs offer a non-cancerous, expandable, and robust human cell source for cell-based BoNT/A1 detection assays using standard and engineered cell culture platforms. Importantly, iPSC-NSCs cultured on synthetic PEG hydrogels generate neural populations with the functional properties required for BoNT uptake and eventual SNARE cleavage (Fig. 1), achieving sensitivities for detecting active BoNT/A1 that were comparable to benchmark EC50 values previously reported for cell-based assays^8^,^9^,^10^,^11^,^12^,^13^,^14^,^15^,^16^ and an approved animal testing protocol for safety assessment. Matrigel is commonly used to promote 3D cellular self-assembly into model tissues *in vitro*^65^, but introduces uncertainty due to a composition that includes numerous bioactive components such as growth factors and extracellular matrix proteins^55^. In contrast, PEG hydrogels promoted iPSC-NSC differentiation and 3D self-assembly into model neural tissues despite presenting minimal bioactive cues (only CRGDS adhesion peptide). Thus, the inherent capacity for *in vitro* self-assembly of human iPSC-NSCs into functional neuronal tissues can be harnessed using a minimally complex, synthetic culture platform. Further, our protocol was developed using monolayer culture techniques that are directly translatable to quantitative and/or enhanced throughput screening approaches^50^, which is a particular advantage compared to culture models that require suspension culture or 3D seeding in Matrigel^65^. Thus, human pluripotent stem cell-derived neuronal cell types cultured on chemically-defined hydrogels offer an alternative to standard platforms for cell-based neurotoxicity screening.

## Methods

### Poly(ethylene glycol) (PEG) Hydrogels

PEG hydrogels were formed using “thiol-ene” photopolymerization to crosslink 8-arm PEG-norbornene (PEG-NB) molecules (20000 MW, JenKem USA, 8ARM (TP)-NB-20K) with PEG-dithiol molecules (3400 MW, Laysan Biosciences, SH-PEG-SH-3400)^28^ or a matrix metalloproteinase (MMP)-degradable peptide (KCGGPQGIAGQGCK; Genscript, >90% purity, C-terminus amidated)^57^. To promote cell adhesion, 3–4 mM CRGDS^52^ (Genscript, >90% purity, C-terminus amidated) was incorporated through the thiol of a terminal cysteine group. Frozen stock solutions of 8-arm PEG-NB were prepared at a final concentration of 300 mg/mL by dissolving 300 mg 8-arm PEG-NB solid in 0.8 mL PBS (the lower PBS volume accounts for volume of dissolved solid), which was then sterile filtered using a 0.2 μm nylon syringe filter (Fisher). Stock ~95 mM SH-PEG-SH crosslinker (~190 mM thiol groups), MMP-peptide (~75 mM peptide/150 mM SH), and CRGDS peptide (~100 mM) solutions were prepared and sterile filtered through a 0.22 μm syringe filter (low protein binding PVDF, Millex) before verifying the final concentration by Elman’s assay (Thermo Scientific; PBS used to dissolve all reagents).

Unless otherwise noted, comparisons to tissue culture polystyrene used a non-degradable PEG-dithiol formulation with 40 mg/mL 8-arm PEG-norbornene (16 mM norbornene arms), 50% SH-PEG-SH crosslinking density (8 mM SH, 50% mol fraction relative to norbornene arms), 3 mM CRGDS, and 0.05% (wt/wt) Irgacure 2959 (I2959 photoinitiator). PEG hydrogels were formed by pipetting 8 μL monomer into 96-well roundbottom plates (TPP® tissue culture plates, Sigma-Aldrich) or 10 μL into 96-well angiogenesis plates (μ-Plate Angiogenesis ibiTreat, Ibidi) and exposing to UV light (~365 nm, Top Shelf UVP XX-15L lamp/stand, Fisher) for two minutes. PEG hydrogels were formed in 96-well roundbottom plates for immunofluorescence imaging or neurotoxicity screening and angiogenesis plates for immunofluorescence imaging to limit meniscus formation and to prevent hydrogel buckling due to confinement during swelling (overnight incubation in basal medium, 37 °C, 5% CO_2_).

### Cell culture

All cells were maintained at 37 °C and 5% CO_2_. Human induced pluripotent stem cell-derived neurons (iCell Neurons, Cellular Dynamics Inc) were cultured according to manufacturer’s protocols, as previously described in detail^11^. Briefly, iPSC-neurons were seeded at a density of 35,000–40,0000 cells/cm^2^ on hydrogels or poly-L-ornithine (PLO; Sigma-Aldrich) and Matrigel (BD Biosciences) coated tissue culture plates (96-well, TPP, MidSci) and cultured for one week using media provided by the manufacturer before neurotoxicity screening.

Human induced pluripotent stem cell-derived neural stem cells (HIP^TM^ Neural Stem Cells, BC1 line, referred to as ‘iPSC-NSCs’) and all associated media components for the manufacturer’s protocol were provided by MTI-GlobalStem, Inc. *Expansion Medium*: Neurobasal® medium (Life Technologies) with 2% B-27® supplement (Life Technologies), 2 mM (1X) Glutagro (L‐ Alanine/L‐Glutamine dipeptide, Corning), 1X MEM non-essential amino acids (Corning), and 20 ng/mL FGF2 (GlobalStem). *Differentiation Medium*: Glasgow’s MEM (Life Technologies) supplemented with 10% KnockOut serum replacement (Life Technologies), 100 μM beta-mercaptoethanol (Life Technologies), and 200 μM sodium pyruvate (Life Technologies), and conditioned using a proprietary protocol (GlobalStem). *Maintenance Medium*: NeuralQ™ Basal Medium (GlobalStem) with 2% GS21 Supplement (GlobalStem) and 0.5 mM Glutagro. To optimize BoNT/A1 sensitivity, the protocol described here was modified from the manufacturer’s recommendation for differentiating iPSC-NSCs into neurons. For optimization, iPSC-NSCs were expanded and differentiated according to manufacturer’s instructions or modified as follows: **(1)** Cells were plated on either laminin or Matrigel coated plates (8.3 μg/cm^2^) and matured for 1–4 weeks. **(2)** Plates were first treated with 0.01% poly-L-ornithine (PLO) before laminin or Matrigel coating instead of the manufacturer’s proprietary coating solution. **(3)** Cells were differentiated in the presence of 1–10 μM retinoic acid (RA) and 5 μM purmorphamine (PUR). **(4)** Cells were plated onto PLO/laminin coated TCP after expansion, and then differentiated and matured on the same plate rather than first differentiating in a flask and then transferring to a new plate. **(5)** Cells were seeded at densities ranging from 5,000 cells per well to 20,000 cells per well. **(6)** The proprietary differentiation and/or maintenance medium were replaced with Neurobasal medium supplemented with B27, glutamax, and with or without additional factors including cAMP, BDNF, GDNF. We note that the manufacturer has since modified the protocol for neural differentiation of iPSC-NSCs that was used as the control condition in the present study.

#### Optimized protocol for differentiation and maturation of iPSC-NSCs

Cryopreserved iPSC-NSCs were plated on a TCP flask coated with 8.3 μg/cm^2^ growth factor reduced Matrigel (BD Biosciences; 1 hr. at 37 °C in expansion medium) and cultured in expansion medium until confluent. Cells were detached using Accutase and seeded in expansion medium at a density of 10,000 cells per well of a 96-well plate (Techno Plastic Products [TPP], MidSci) that was pre-coated overnight with 0.01% poly-L-ornithine (PLO, Sigma-Aldrich), followed by coating with 15 μg/ml of mouse laminin (Life Technologies) for 1 h at 37 °C. Cells were then cultured for one day in expansion medium, and an additional five days in differentiation medium containing 2 μM retinoic acid (“RA”, Stemgent, Inc.) and 5 μM purmorphamine (“PUR”, Calbiochem). A fresh RA aliquot was used for each differentiation (stock concentration = 10 mg/mL in DMSO; store at −20 °C in the dark). Cells were matured in maintenance medium (without detachment) and matured for up to 23 days prior to use in a toxin assay.

For experiments on PEG hydrogels, iPSC-NSCs were first expanded to confluence on standard plates in expansion medium (as described above). The iPSC-NSCs were detached using Accutase, seeded on PEG hydrogels at a density of 50,000 cells/well, and cultured in expansion medium until confluent (2–3 days). Upon confluence, iPSC-NSCs were cultured on PEG hydrogels for five days in differentiation medium with or without 2 μM retinoic acid and 5 μM purmorphamine (as indicated), with half of the medium exchanged every two days. Cells were then matured in maintenance medium for up to 23 days prior to use in a toxin assay (as indicated), with about half of the medium exchanged every 3–5 days. iPSC-NSCs cultured on PEG hydrogels and differentiated in the presence of RA and PUR could be maintained for over three months without an apparent change in cell morphology.

### Botulinum neurotoxin assay

Pure Botulinum neurotoxin A (BoNT/A) was prepared from *Clostridium botulinum* strain Hall A hyper as previously described^66^. The toxin was dissolved in phosphate buffered saline, pH 7.4 and 40% glycerol, and stored at −20 °C until use. Activity of the BoNT/A1 preparation was determined by the mouse bioassay^53^,^54^, and specific toxicity was about 1.25 × 10^8^ mouse LD_50_ Units/mg (U/mg). After the indicated maturation times, cells were exposed to serial dilutions of BoNT/A1 in maintenance medium for 48 h at 37 °C, 5% CO_2_. The toxin was then aspirated and cells lysed in 50 μl of 1× LDS lysis buffer (Invitrogen). The samples were analyzed by Western blot essentially as described previously^16^,^67^, using a monoclonal anti-SNAP-25 antibody (Synaptic Systems, Germany) and secondary anti-mouse AP conjugated antibody (KPL). Bands were visualized with PhosphaGlo chemiluminescent reagent (KPL) and densitometry was done using a Foto/Analyst FX system and TotalLab Quant software (Fotodyne). Data plots and best-fit lines (four parameters – variable slope) were generated and EC50 values estimated using PRISM 6 software.

### Gene Expression by Quantitative Reverse Transcription-PCR

Total RNA was isolated using RNEasy Plus (Qiagen). 100 ng of total RNA was reverse-transcribed using the qScript cDNA Synthesis Kit (Quanta Biosciences). Real-time PCR was performed using PerfeCTa SYBR Green Supermix (Quanta Biosciences) and 300 micromolar primers in an Mx3005P Real-Time instrument (Agilent) for 40 cycles of 95 °C/15 seconds, 58 °C/20 seconds, 72 °C/30 seconds, followed by melt curve analysis. The relative amounts of PCR product were quantified using the relative threshold cycle (ΔΔCt) method. The gene quantities for each sample were normalized against *GAPDH* (glyceraldehyde-3-phosphate dehydrogenase). See Table 1 for the full list of primers used in the present study.

### Immunofluorescence Imaging

*Blocking buffer*: 0.25% Triton X-100 and 1% BSA in PBS. *Incubation buffer*: 0.05% Triton X-100 and 1% BSA in PBS. *Rinse buffer*: 0.05% Triton X-100 in PBS. See Table 2 for the full list of antibodies and dilutions used in the present study. Immunostaining for PEG hydrogels was performed using 96-well round bottom (TPP culture plates, Sigma-Aldrich) or angiogenesis plates (μ-Plate Angiogenesis 96 well, ibiTreat, Ibidi). Cells were fixed for 60 min. with 2% buffered formalin and then rinsed with phosphate buffered saline (PBS) before further processing (or stored in PBS at 4 °C until immunostaining). Cells were permeabilized and blocked in blocking buffer (at least 60 min.). Primary antibodies were prepared in incubation buffer (See Table for dilutions), added to the samples, and incubated overnight at 4 °C. Cells were rinsed in rinse buffer (2 × 60 min.) followed by a third rinse step in incubation buffer (at least 60 min.). Secondary antibodies (1:200) and DAPI (1:1000, Sigma-Aldrich) were prepared in incubation buffer, added to the samples, and incubated overnight at 4 °C. Samples were then rinsed 2 × 60 min. in rinse buffer, followed by an additional overnight rinse at 4°C in rinse buffer. Samples were then stored in PBS until imaging (typically at least 24 hours). Confocal images were collected using a Nikon A1R laser scanning confocal microscope with a Plan Apo 10× objective. Images were cropped to 500 × 500 or 750 × 750 μm using ImageJ^68^,^69^.

### Statistical Analysis

Statistical significance was determined by Student’s *t* test for RT-PCR data (Fig. 4). Prism was used to perform a one-way ANOVA (alpha = 0.05) followed by a Tukey test to compare individual means for EC50 data (Figs 2, 3, Supplementary Fig. S1). The EC50 values from Fig. 3 and Supplementary Fig. S1 were combined for statistical analysis (see Supplementary Table S1 for all sample comparisons).

## Additional Information

**How to cite this article**: Pellett, S. *et al.* Human Induced Pluripotent Stem Cell Derived Neuronal Cells Cultured on Chemically-Defined Hydrogels for Sensitive *In Vitro* Detection of Botulinum Neurotoxin. *Sci. Rep.* **5**, 14566; doi: 10.1038/srep14566 (2015).

## Supplementary Material

Supplementary Figure S1

Supplementary Table S1

## Figures and Tables

**Table 1:** List of RT-PCR primers.

Gene	Forward Primer (5′-3′)	Reverse Primer (5′-3′)
vGluT1	TACACGGCTCCTTTTTCTGG	CTGAGGGGATCAGCATGTTT
SLC18A3	CATCGCCGACATCTCCTATT	AGCAAGACGGGAGCATAGAG
SLC32A1	CACGACAAGCCCAAAATCAC	AGAAACAACCCCAGGTAGCC
ChAT	AAAAGGTCCCCCGTAAGATG	TGCTCCTCAGACACCAAGTG
DBH	TCTCGGCACCACATTATCAA	TCGGGTTTCATCTTGGAGTC
GAD67	CCTGGAACTGGCTGAATACC	CCCTGAGGCTTTGTGGAATA
vGluT2	ATTCCATCAGCAGCCAGAGT	AGGAGGTGGTTGCCAGTCTA
GFAP	GGTTGAGAGGGACAATCTGG	CAGCCTCAGGTTGGTTTCAT
RBFOX3	CCGAGTGATGACCAACAAGA	GAATTCAGGCCCGTAGACTG
TuB3	GGCCTTTGGACATCTCTTCA	CCTCCGTGTAGTGACCCTTG

**Table 2:** List of antibodies used for immunofluorescence imaging.

Primary	Antibody	Vendor	Catalog #
1:500	Polyclonal Rabbit Anti-Glial Fibrillary Acidic Protein (GFAP)	DAKO	Z033401-2
1:500	Mouse Anti-Neuron-specific β3-Tubulin MAb (Clone TuJ-1)	R&D Systems	MAB1195
1:200	Mouse Anti-Anti-MAP2 Antibody, clone AP20	Millipore	MAB3418
1:500	Rabbit Anti-GABA	Abcam	ab43865
1:50-1:100	Rabbit Anti-VGLUT2	Abcam	ab84103
1:250	Polyclonal rabbit antibody, Synaptotagmin 2, cytoplasmic domain (SYT2)	Synaptic Systems	105 123
1:100	Rabbit Anti-Glutamate Decarboxylase 65 & 67 Antibody (GAD 65/67)	EMD Millipore	AB1511
1:100	Synaptophysin (D35E4) XP® Rabbit mAb #5461	Cell Signaling	5461S
1:100	Synapsin-1 (D12G5) XP® Rabbit mAb #5297	Cell Signaling	5297S
1:100	Mouse anti-MNR2/HB9/Mnx1	DSHB	81.5C10
**Secondary**	**Antibody**	**Vendor**	**Catalog #**
1:200	Alexa Fluor® 568 Donkey Anti-Mouse IgG	Life Technologies	A10037
1:200	Alexa Fluor® 568 Donkey Anti-Rabbit IgG Antibody	Life Technologies	A10042
1:200	Alexa Fluor® 488 Donkey Anti-Mouse IgG (H+L) Antibody	Life Technologies	A-21202
1:200	Alexa Fluor® 488 Donkey Anti-Rabbit IgG (H+L) Antibody	Life Technologies	A-21206
