# Supplementary material for: Human Induced Pluripotent Stem Cell Derived Neuronal Cells Cultured on Chemically-Defined Hydrogels for Sensitive In Vitro Detection of Botulinum Neurotoxin
Source: Sci Rep. 2015 Sep 28;5:14566. doi: 10.1038/srep14566 (PMC4585966; doi:10.1038/srep14566)
Supplement: Supplementary Figure S1 [file srep14566-s1.pdf]

Supplementary Figure

Human Induced Pluripotent Stem Cell Derived Neuronal Cells Cultured on Chemically-Defined Hydrogels for Sensitive *In Vitro* Detection of Botulinum Neurotoxin

Sabine Pellett[1,*], Michael P. Schwartz[2,*], William H. Tepp[1], Richard Josephson[3], Jacob M. Scherf[1], Christina L. Pier[1], James A. Thomson[4-6], William L. Murphy[2,7], Eric A. Johnson[1]

[1] Department of Bacteriology, University of Wisconsin at Madison, Madison, Wisconsin, United States of America

[2] Department of Biomedical Engineering, University of Wisconsin at Madison, Madison, Wisconsin, United States of America

[3] MTI-GlobalStem, Inc. Gaithersburg, Maryland, United States of America

[4] Department of Cell and Regenerative Biology, University of Wisconsin at Madison, Madison, Wisconsin, United States of America

[5] Morgridge Institute for Research, Madison, Wisconsin, United States of America

[6] Department of Molecular, Cellular, and Developmental Biology, University of California at Santa Barbara, Santa Barbara, California, United States of America

[7] Department of Orthopedics and Rehabilitation, University of Wisconsin at Madison, Madison, Wisconsin, United States of America

* sludwig@wisc.edu, mpschwartz@wisc.edu

Keywords: tissue engineering, differentiation, toxicology, neurons, cell-based screening

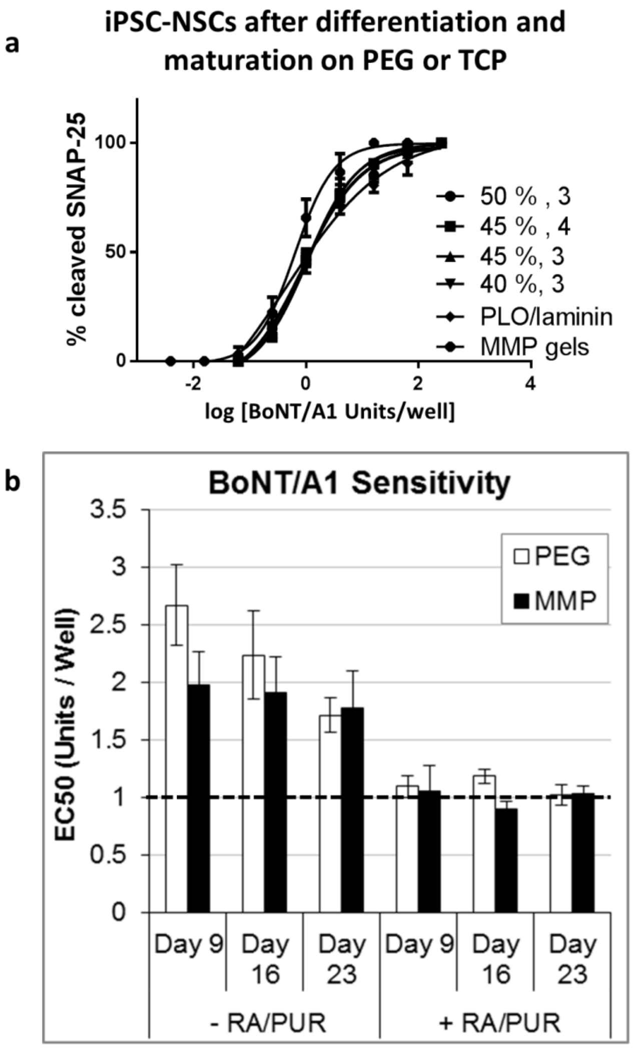

Supplementary Figure S1. BoNT/A1 sensitivity for human iPSC-derived neural stem cells (iPSC-NSCs) cultured on PEG hydrogels and tissue culture polystyrene (TCP). **(a)** Representative EC50 curves for human iPSC-neural stem cells (iPSC-NSCs) treated with serial dilutions of BoNT/A1 (Units/Well). The iPSC-NSCs were differentiated for 5 days (with RA/PUR) and matured on PLO/LAM treated TCP surfaces, hydrogels crosslinked with non-degradable SH-PEG-SH (40-50% crosslinking density) and either 3 or 4 mM CRGDS, or hydrogels crosslinked with a matrix metalloproteinase (MMP)-degradable peptide (50% crosslinking density, 3 mM CRGDS). Cultured cells were exposed to BoNT/A1 for 48 h before cell lysates were harvested, followed by Western blot and densitometry analysis to quantify SNAP-25 cleavage. **(b)** BoNT/A1 sensitivities (EC50, Mean ± S.D., 3 replicate experiments) for iPSC-NSCs that were cultured on PEG hydrogels crosslinked with non-degradable SH-PEG-SH ("PEG": 50% crosslinking density, 3 mM CRGDS) or MMP-degradable peptide ("MMP": 50% crosslinking, 3 mM CRGDS), differentiated for 5 days with (+) or without (-) RA/PUR, and matured for 9, 16, or 23 days. The EC50 value is defined as BoNT activity (Units/Well) required to reach half the maximum response for SNAP-25 cleavage, where 1 U is equivalent to the mLD50 determined using an *in vivo* mouse bioassay (dashed line). See Supplementary Table S1 for statistical analysis using a one-way ANOVA (alpha = 0.05) followed by a Tukey test to compare individual means for TCP, PEG hydrogels, and MMP hydrogels (± RA/PUR; Days 9,16, and 23).
